# Supplementary material for: Extended Depth of Focus Two-Photon Light-Sheet Microscopy for In Vivo Fluorescence Imaging of Large Multicellular Organisms at Cellular Resolution
Source: Int J Mol Sci. 2023 Jun 15;24(12):10186. doi: 10.3390/ijms241210186 (PMC10298976; doi:10.3390/ijms241210186)
Supplement: Supplementary file 1 [file ijms-24-10186-s001.zip › ijms-2432536-supplementary.pdf]

Supplementary Figures for
Extended Depth of Focus Two-photon Light-sheet Microscopy for In Vivo Fluorescence Imaging of Large Multicellular Organisms at Cellular Resolution

Takashi Saitou and Takeshi Imamura

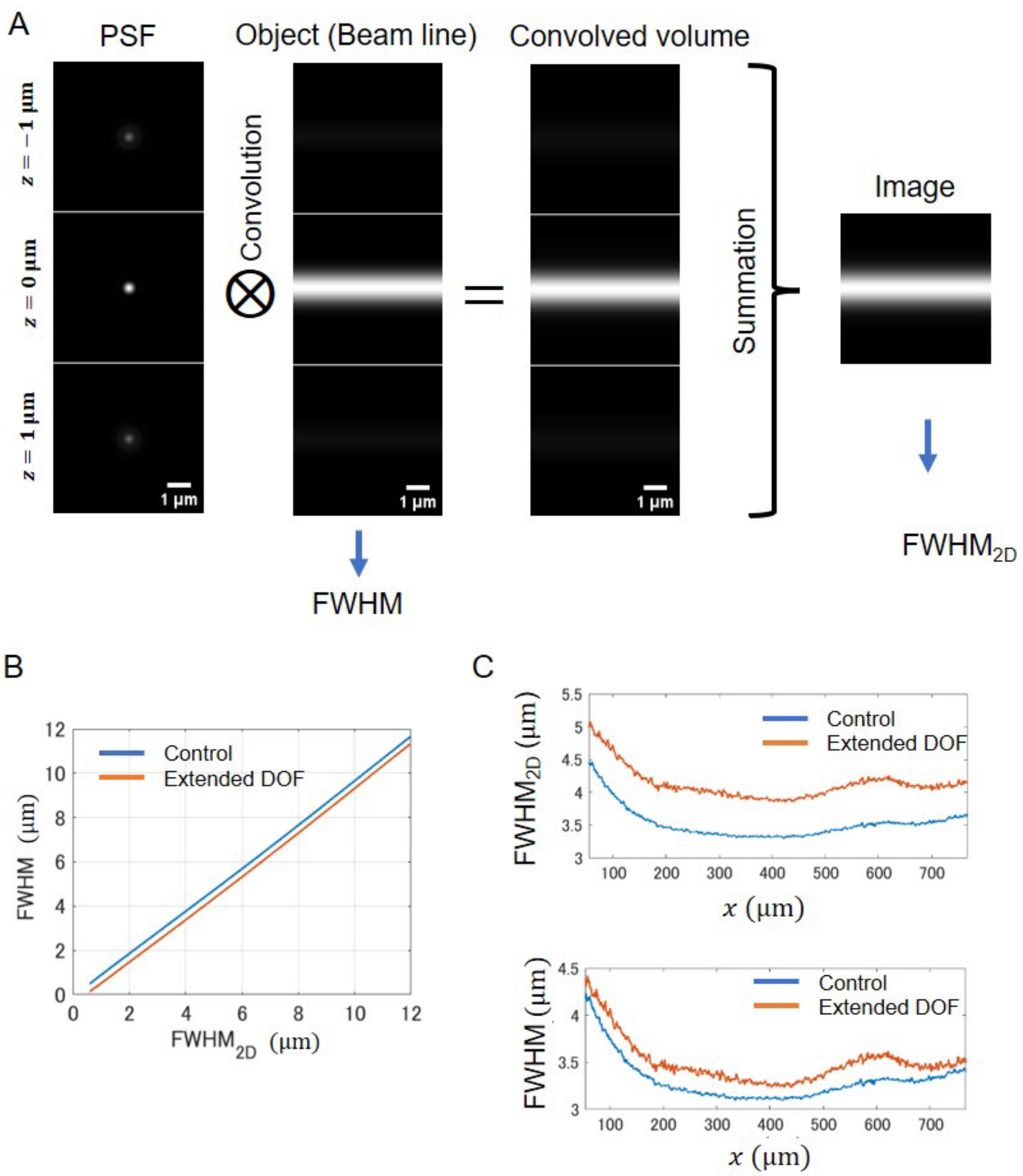

Figure S1. Estimation method of the real FWHM of the illumination beam. (A) A scheme of the calculation. PSFs were calculated by the Debye theory. A 3D observed image is created through 3D convolution of the PSF and object. The acquired 2D image is a sum of the 3D image along the *z*-axis. From the relationship between the 3D object and 2D image, the real FWHM can be estimated. (B) The plot of the FWHM as a function of $FWHM_{2D}$. (C) Measured $FWHM_{2D}$ and estimated FWHM.

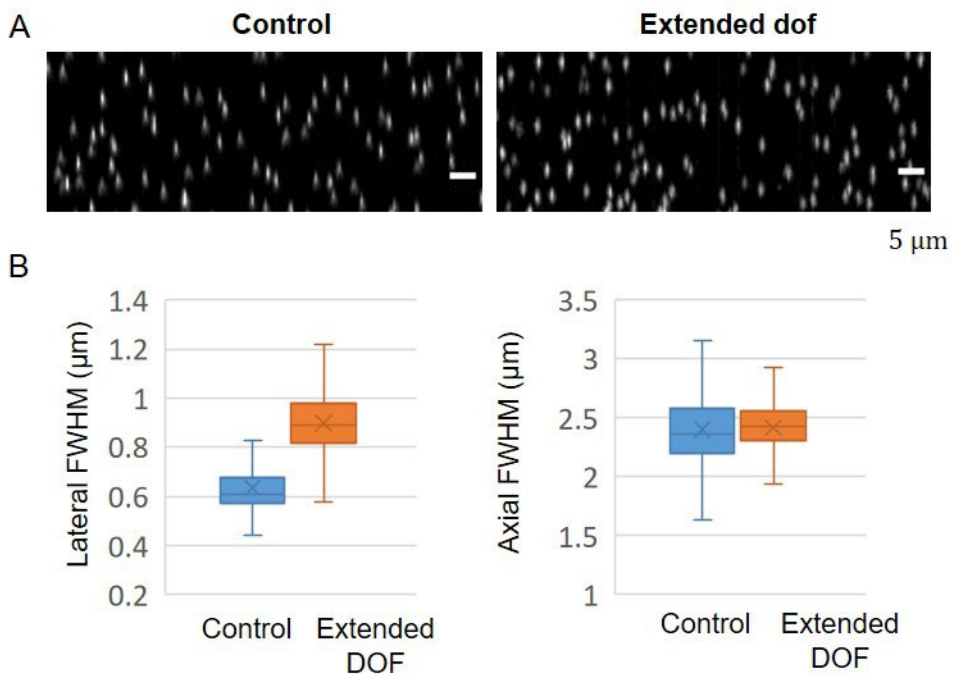

Figure S2. Measurement of the beads PSFs for the full-field images. (A) Fluorescent bead images were measured by the system with and without the device. Maximum intensity projection along the *y* direction is performed for visualization on the *xz* plane. (B) The calculated FWHM values for the lateral and axial directions. The PSF calculation employed over 100 independent beads as data points. In the box plots, the box lines indicate the first quantile, median, and third quantile. Lower and upper whiskers indicate the minimum and maximum, respectively. The cross mark represents the average.
